# Osteoporosis in Pre-menopausal Patients With Anorexia Nervosa: Management, Challenges and Review of Literature

**DOI:** 10.7759/cureus.93659

**Published:** 2025-10-01

**Authors:** Shruthi Rayen, Manjusha Rathi

**Affiliations:** 1 Endocrinology, Sandwell and West Birmingham Hospitals, Birmingham, GBR

**Keywords:** anorexia nervosa (an), bone mineral density, osteoporosis, pre-menopause, relationship between diseases and nutrition

## Abstract

Anorexia nervosa (AN) is a group of psychiatric disorders identified by severe calorie restriction and low body mass index (BMI), which can lead to complications such as osteoporosis. Osteoporosis in AN patients can be particularly challenging to manage as it affects majorly a young and pre-menopausal demographic (most commonly diagnosed when under 25 years of age) and lack of clear pharmacological guidelines within this population. We present the case of a 39-year-old woman with a long-standing history of AN who was diagnosed with osteoporosis through dual energy X-ray absorptiometry (DEXA) scan. Over the subsequent years, pharmacological treatments were trialed; however, many were stopped either due to unwanted side effects or concerns regarding long-term risks in a young woman. Eventually, the consensus was reached to focus on improving her nutrition and weight gain, which remains the best-evidenced method of improving bone mineral density in patients with AN. This case highlights the difficulties in treating osteoporosis in premenopausal patients with AN and indicates the need for more research in this population and clear pharmacological guidelines for healthcare professionals.

## Introduction

Anorexia nervosa (AN) describes a psychiatric disorder characterised by fear of weight gain and body dysmorphia resulting in extreme calorie restriction, and subsequently low body mass index (BMI). AN is associated with numerous physiological complications and has the highest mortality rate of any psychiatric disorder, resulting from chronic malnutrition. Osteoporosis is a common complication that can affect up to 50% of AN patients [1]. Osteoporosis is a condition characterised by low bone mineral density (BMD) and is generally treated with anti-resorptive drugs, anabolic agents, hormone replacement therapy (HRT), biologics and vitamin supplementation. However, treating osteoporosis in the AN patient population presents a particular challenge. There are no current guidelines pertaining to the pharmacological management of osteoporosis in AN patients, the majority of whom are young premenopausal women. Bisphosphonates should be used with caution in women of childbearing age due to teratogenic effects, and similarly commencing HRT can pose many risks in this population. As osteoporosis in AN patients is difficult to treat, the consensus remains that restoration of weight and return to regular menses are the strongest factors for increasing BMD [2]. This case report outlines the current best practice for healthcare professionals and highlights the need for further research into pharmacological interventions in this population.

## Case presentation

We present the case of a 39-year-old woman with a medical history significant for AN, mixed anxiety and depression disorder, fibromyalgia, iron-deficiency anaemia, and temporomandibular joint pain syndrome. She was referred to secondary care endocrinology for the management of osteoporosis identified on dual-energy X-ray absorptiometry (DEXA) scan. There was no history of low-trauma fractures; she was a non-smoker and did not consume alcohol. Apart from AN, she did not have any other obvious secondary cause, which may contribute to low bone mineral density, such as hyperthyroidism, primary ovarian insufficiency, exposure to anti-epileptic medication or corticosteroid use.

Her initial relevant presentation was a 2011 hospital admission under gastroenterology prompted by significant weight loss of over 30 kg in three years (65 kg in 2008 to 31.2 kg in 2011). She declined a trial of nasogastric feeding and self-discharged against medical advice. One year later, she had an unresponsive episode. On arrival to the hospital, her capillary blood glucose level was 1.1 mmol/L (reference range 4-7 mmol/L), and she displayed physical signs and stigmata of severe malnourishment and cachexia. She received prompt medical treatment and was provided with a clear and careful plan for ongoing weight gain by dieticians. Subsequent psychiatric review demonstrated no evidence of other psychiatric illnesses. It was on discharge from this admission that a DEXA bone density scan was performed (Table 1), confirming a diagnosis of severely low bone mineral density for age secondary to AN, as evidenced by a Z-score <2.0, prompting commencement of a bisphosphate (alendronate) and a calcium/vitamin D tablet (Adcal-D3).

**Table 1:** The patient’s dual-energy X-ray absorptiometry (DEXA) scan results over time. Anterior-posterior spine/neck of femur T score more than -1.0 considered normal and less than -2.5 indicates osteoporosis. Anterior-posterior spine Z score/neck of femur Z score more than -2.0 considered normal and less than 2.0 indicates low bone mineral density for what is expected of your age. As this patient was premenopausal, Z score was used to diagnose severely low bone density for age secondary to anorexia nervosa.

T score and Z score	Year of result		
	April 2012	May 2018	June 2021
Anterior-posterior spine T score	-4.3	-4.0	-3.9
Neck of femur T score	-3.6	-3.2	-4.3
Anterior-posterior spine Z score	-4.3	-2.8	-2.7
Dual femur Z score	-3.6	-2.9	-3.4

Six years later in 2018, this patient was referred to endocrinology services for assessment of her osteoporosis and multiple year history of amenorrhea (Table 2). Her BMI was now 12.2 kg/m^2^ (reference range for healthy weight 18.5-24.9kg/m^2^), and she was suffering from significant mobility issues. The initial management plan involved substituting alendronate for six-monthly subcutaneous denosumab 60 mg (a monoclonal antibody used in osteoporosis treatment), commencing HRT, and planned follow-up in nine months.

**Table 2:** The patient’s blood results at the 2018 endocrine review

Blood Test	Result	Reference ranges
Haemoglobin A1c	33 mmol/mol	<48 mmol/mol
Bone Profile	Normal	-
Thyroid-Stimulating Hormone	0.98 mU/L	0.4-4 mU/L
Liver Function Tests	Normal	-
Glomerular Filtration Rate	>90 mL/min/1.73m^2 ^	>90 mL/min/1.73m^2^
Urea and Electrolytes	Normal	-
Vitamin B12	450 pg/ml	>350 pg/ml
Folate	6.7 ng/ml	>4 ng/ml
Follicle-Stimulating Hormone	3 IU/L	Follicular phase 1-9 IU/L; Ovulatory phase 6-26 IU/L; Luteal phase 1-9 IU/L
Luteinising Hormone	<1 IU/L	Follicular phase 1-12 IU/L; Ovulatory phase 16-104 IU/L; Luteal phase 1-12 IU/L
Oestradiol	<37 pmol/L	80-1400 pmol/L
Tissue Transglutaminase	<1.9 U/ml	<7 U/ml = negative; >10 U/ml = positive
Cortisol (10:46 a.m.)	408 nmol/L	<150 nmol/L = adrenal insufficiency

However, in May 2019 the patient - now BMI 11.3 kg/m^2^ - was re-reviewed earlier than planned due to concerns regarding the denosumab injections and the gastro-intestinal side effects of oral HRT preparation, predominantly dyspepsia. To mitigate these unwanted side effects, she was switched back to alendronate and commenced on twice-weekly HRT patch instead of oral tablet. In November 2019, at BMI 12 kg/m^2^, the patient reported nausea, vomiting, and fatigue with bi-weekly patches and ongoing gastrointestinal side effects of nausea and vomiting. An alternative anti-resorptive bone protection agent, Raloxifene, was suggested; however, the common side effect of leg cramps was deemed unsuitable by the patient due to concurrent diagnosis of fibromyalgia. The decision to continue with alendronic acid alone was agreed.

This case was discussed in a multidisciplinary endocrine meeting in June 2020. The consensus was reached that adequate nutrition remained the mainstay treatment for her osteoporosis. Alendronic acid was held for one year with a plan for repeat DEXA scan and follow-up at this time. The final endocrinology patient review was in done June 2021, and the patient had not sustained any fractures in this interval of time. At the patient’s request, she was discharged back to her general physician (GP) with emphasis that weight restoration was going to be the optimal option for her osteoporosis management.

## Discussion

AN can cause severe and complex physiological effects, which mostly stem from the consequences of malnutrition. Development of osteoporosis in AN patients has genetic, intrinsic, extrinsic and lifestyle factors contributing to the risk of disease [3]. Loss of BMD has been shown to result from low weight, reduced adiposity, low gonadal function, excess glucocorticoid levels, and an impaired insulin-like growth factor axis [2,4].

Multiple studies have demonstrated the efficacy of bisphosphonates in improving BMD, particularly spinal BMD which is the main area of bone density loss in anorexia nervosa patients [5]. As such, they are often initiated in patients with AN and osteoporosis, as demonstrated in our case above. However, the majority of these studies have been performed on perimenopausal, or postmenopausal, women. There is a paucity of data on the effects of these medications in pre-menopausal women such as our subject. Indeed, bisphosphonates are not licensed for use in this cohort, and they should be used with caution in young women of reproductive age owing to their long half-life, accumulation in maternal tissue, and potential teratogenic effects - requiring pregnancy counselling and contraceptive advice [4].

Denosumab is another drug sometimes used for the treatment of osteoporosis in postmenopausal women. There is limited but intriguing evidence for its use in patients with AN. One study looking at three female patients with AN saw markedly decreased bone turnover markers with denosumab over 24 months [6]. Another study investigated the off-label use of denosumab in AN patients and found reduced bone turnover and increased spine BMD after 12 months [7]. As denosumab has a shorter half-life and does not demonstrate the same level of skeletal accumulation compared to bisphosphonates, it may seem a preferable alternative to bisphosphonate use. However, the efficacy and safety of the medication has not been defined on a large scale in premenopausal women.

Hormone replacement therapy (HRT) is often used to preserve BMD in post-menopausal women. A systematic review looking at the effects of HRT in peri- and premenopausal women demonstrated good evidence for a positive effect of oral contraceptives on BMD in perimenopausal women, and fair evidence in “hypothalamic” oligomenorrhoeic and amenorrhoeic premenopausal women, but limited evidence for a positive effect in anorexic but otherwise healthy premenopausal women [8]. With limited evidence supporting the use of HRT in premenopausal anorexic women, it can be difficult to justify its use given the many and common side effects experienced by patients.

Other drugs such as teriparatide and raloxifene are licensed for the treatment and prevention of postmenopausal osteoporosis, but not for treatment in the demographic we are scrutinising. With studies suggesting that weight restoration in AN patients is the most important determinant in restoration of BMD, this ends up being a final refuge for the management of osteoporosis, as was the case for the patient described above [9]. The benefit of weight gain on BMD is augmented by return of menses and improvement in gonadal function, which may even improve BMD independently of weight gain [10].

## Conclusions

The current guidelines on treatment of osteoporosis, including osteoporosis secondary to AN, are based on research and literature focusing on post-menopausal women. As most AN patients are premenopausal, pharmacological treatment options unfortunately remain flawed and there is a clear lack of evidence-based guidelines for practitioners to refer to. The best-evidenced approach in this population is currently with weight gain and restoration of regular menses. Due to the many difficulties in achieving weight gain, and as loss of BMD can cause irreparable damage to patients, there is a need for large-scale research on the use of anti-osteoporotic agents in the premenopausal female population. This would help form guidance on the management of AN patients who develop osteoporosis.
